# Anti-GD2 Based Immunotherapy Prevents Late Events in High-Risk Neuroblastoma Patients over 18 Months at Diagnosis

**DOI:** 10.3390/cancers13194941

**Published:** 2021-09-30

**Authors:** Michelle L. Tas, Lisa W. Dootjes, Marta Fiocco, Ronald R. de Krijger, Miranda P. Dierselhuis, Natasha K. A. van Eijkelenburg, Martine van Grotel, Kathelijne C. J. M. Kraal, Annemarie M. L. Peek, Godelieve A. M. Tytgat, Max M. van Noesel

**Affiliations:** 1 Department of Solid Tumors, Princess Máxima Center for Pediatric Oncology, 3584 CS Utrecht, The Netherlands; m.tas@prinsesmaximacentrum.nl (M.L.T.); lisadootjes@gmail.com (L.W.D.); R.R.deKrijger-2@prinsesmaximacentrum.nl (R.R.d.K.); M.P.Dierselhuis@prinsesmaximacentrum.nl (M.P.D.); N.K.A.vanEijkelenburg@prinsesmaximacentrum.nl (N.K.A.v.E.); M.vanGrotel@prinsesmaximacentrum.nl (M.v.G.); K.C.J.Kraal@prinsesmaximacentrum.nl (K.C.J.M.K.); a.m.l.peek@prinsesmaximacentrum.nl (A.M.L.P.); G.A.M.Tytgat@prinsesmaximacentrum.nl (G.A.M.T.); 2 Department of Pediatric Oncology, Erasmus Medical Center, 3015 CN Rotterdam, The Netherlands; 3 Princess Máxima Center for Pediatric Oncology, Trial and Data Center, 3584 CS Utrecht, The Netherlands; M.F.Fiocco@prinsesmaximacentrum.nl; 4 Mathematical Institute, Leiden University, 2333 CA Leiden, The Netherlands; 5 Department of Biomedical Data Science Section Medical Statistics, Leiden University Medical Center, 2333 ZA Leiden, The Netherlands; 6 Department of Pathology, University Medical Center Utrecht, 3508 GA Utrecht, The Netherlands; 7 Department of Cancer and Imaging, University Medical Center Utrecht, 3584 CX Utrecht, The Netherlands

**Keywords:** neuroblastoma, high-risk, metastatic, immunotherapy, anti-GD2

## Abstract

**Simple Summary:**

High-risk neuroblastoma accounts for 4% of newly diagnosed pediatric malignancies, but for 9–10% of pediatric cancer mortality. To reduce the number of (late) recurrences and subsequently improve survival, anti-GD2 monoclonal antibody based immunotherapy has been added to the maintenance phase of treatment. The first randomized study (ANBL0032) was ground breaking, showing a 20% improved event free survival. Subsequently immunotherapy was included in all international high-risk treatment regimens. Randomization will never be repeated. In this article we present additional data from our retrospective cohort to corroborate the ANBL0032 study. Our cohort contains 84 Dutch high-risk neuroblastoma patients. They were treated with GPOH or POG induction, followed by immunotherapy according to original ANBL0032 protocol (immunotherapy group) or single-agent isotretinoin (historical control group). In the complete cohort, 5 year OS was 64 ± 7% and 49 ± 8% for the immunotherapy group and the control group, respectively (*p* = 0.16). Five year EFS was 57 ± 7% and 41 ± 8%, respectively (*p* = 0.16). In the subgroup of patients ≥ 18 months, 5-yr OS was 63 ± 8% and 39 ± 9, respectively (*p* = 0.04) and EFS 54 ± 8% and 29 ± 8%, respectively (*p* = 0.05). Our five year data suggest a role for the immunotherapy in preventing late events, especially in patients ≥ 18 months old.

**Abstract:**

Background: Anti-GD2 based immunotherapy has improved overall (OS) and event free survival (EFS) for high-risk neuroblastoma (HR-NBL) patients. Here, we evaluate the long-term efficacy of anti-GD2 immunotherapy in combination with isotretinoin, GM-CSF, and IL-2. Methods: Dutch HR-NBL patients treated with immunotherapy according to the COG-ANBL0032 protocol (*n* = 47) were included and compared to historical controls (*n* = 37) treated with single-agent isotretinoin maintenance therapy. Survival time was calculated from start of the maintenance therapy. Results: The study and control group were similar concerning baseline characteristics. In the complete cohort, 5 year OS was 64 ± 7% and 49 ± 8% for the immunotherapy group and the control group, respectively (*p* = 0.16). Five year EFS was 57 ± 7% and 41 ± 8%, respectively (*p* = 0.16). In the subgroup of patients ≥ 18 months, 5-yr OS was 63 ± 8% and 39 ± 9, respectively (*p* = 0.04) and EFS 54 ± 8% and 29 ± 8%, respectively (*p* = 0.05). Landmark analysis for EFS with landmark point at 6 months after start of maintenance suggests a larger effect on the prevention of late than early events. Conclusions: This study is the first to confirm the results of the COG-ANBL0032 study in a cohort treated with a different induction regimen. Anti-GD2 immunotherapy prevents late events, most significantly in patients older than 18 months of age at diagnosis.

## 1. Introduction 

Neuroblastoma is a pediatric tumor with an incidence of 11 per million children < 15 years of age each year [1]. It accounts for 7% of newly diagnosed pediatric malignancies, and for 10–12% of pediatric cancer mortality [2,3]. It is a heterogeneous tumor with good survival (>85%) for low-risk patients with minimal treatment, but with poor survival (<50%) for high-risk patients, despite intensive multimodality treatment [4,5]. The main challenges in treating high-risk patients are improving complete response (CR) rates and reducing the number of recurrences after CR to initial treatment: 50–60% of patients will develop recurrent disease, which is associated with 5-yr survival rates below 20% [6,7]. To improve survival, therapy for high-risk neuroblastoma has been intensified in the last decades. Most recently, anti-GD2-based immunotherapy was added to the maintenance phase of treatment [4,8]. Ganglioside-2 (GD2) is a tumor-associated antigen, expressed on 95% of neuroblastoma cells [9]. In normal tissues, expression is limited to the central and peripheral nervous system. Other tumors expressing GD2 include melanoma, glioblastoma multiforme, medulloblastoma, small cell lung carcinoma, breast cancer, and some sarcomas [10,11,12,13].

Between 2001 and 2009, the American Children’s Oncology Group (COG) conducted a randomized controlled trial (ANBL0032) comparing the ch14.18 antibody (dinutuximab) in combination with isotretinoin and alternating GM-CSF and IL-2 to single-agent isotretinoin in the maintenance phase of treatment [3,14]. This improved event free survival (EFS) by 20% and 11%, and overall survival (OS) by 11% and 16% after 2 and 5 years, respectively [3,14]. The authors concluded dinutuximab to be more powerful in preventing early recurrences than late recurrences.

Here, we conducted a retrospective analysis in a Dutch cohort treated according to the COG-ANBL0032 protocol. We present data on long-term survival suggesting a preventive effect on late events, and show that this is the most pronounced in patients ≥ 18 months of age at diagnosis.

## 2. Materials and Methods

### 2.1. Patients Characteristics

High-risk neuroblastoma patients in the Dutch Childhood Oncology Group (DCOG) database, diagnosed between 1999 and 2015, were identified based on completion of induction therapy including consolidation therapy with high-dose chemotherapy and autologous stem cell rescue. Exclusion criteria were similar to the ANBL0032 study: (1) less than partial response (PR) after induction therapy; (2) interval between start of induction therapy and ASCT of more than 12 months; (3) progressive disease before start of the maintenance treatment; and (4) induction therapy according to other protocols than the DCOG NBL2004 [15] or POG9640 [16] protocols. Patients who received immunotherapy according to the ANBL0032 protocol were diagnosed between 2009 and 2015 and were compared to historical controls diagnosed between 1999 and 2014. Five patients in the control group were diagnosed between 2010 and 2014 when immunotherapy was available, for all these cases it was parents’ choice not to receive the immunotherapy. The historical control group was treated with an identical induction regimen and high dose chemotherapy as consolidation. The maintenance treatment was single-agent isotretinoin 160 mg/m^2^/day for subsequent 14 days followed by 14 day rest for a total of 6–9 cycles.

Clinical and pathological characteristics were collected for all patients at diagnosis: age, INSS stage, MYCN status, and LOH1p status. All available tumor samples were centrally reviewed using the International Neuroblastoma Pathology Classification (INPC) [17] by an expert pathologist (RdK). Response after induction treatment was retrieved from patient charts and reconstructed from the available clinical, pathological, radiological, and biochemical data and centrally discussed by MT, LD, GT, and MvN following the 1993 International Neuroblastoma Response Criteria (INRC) [18]. Since all patients were diagnosed before 2017 and assessments from charts were not yet according to the 2017 INRC criteria [19]. Collection of patient data and use of tumor material was approved by the Medical Research Ethics Committee of the University Medical Center Utrecht, Utrecht, The Netherlands (reference number: WAG/nb/18/021561). In addition, all patients who received immunotherapy signed a written consent for the ANBL0032 study.

### 2.2. Statistical Analysis

Data were analyzed using SPSS Statistics 26 (IBM Corp., Armonk, NY, USA, 2019). Fisher’s exact test was used to analyze clinical and pathologic characteristics. Estimated OS and EFS were estimated according to Kaplan–Meier methodology [20] and are reported ±SE. Survival was calculated from start of maintenance treatment to event or last follow-up. Events were defined as recurrence, progression, or death. To investigate the effect of immunotherapy on survival outcomes a Cox regression model was estimated [21]. To deal with the violation of the proportional hazard assumption for treatment, two separate models were estimated [22,23]. The first was estimated from start of the maintenance treatment until the end, 6 months later. The second model was estimated from the landmark point (6 months after start of maintenance) until 5 years after start of maintenance therapy. Multivariable Cox regression estimation was used to estimate the effect of induction protocol on survival. *p* < 0.05 was considered significant for all tests.

## 3. Results

### 3.1. Patient Characteristics

A total of 103 patients was identified who were diagnosed with high-risk neuroblastoma between 1999 and 2015 and had completed induction therapy and consolidation therapy with high-dose chemotherapy followed by autologous stem cell transplantation (ASCT). Nineteen patients were excluded from analysis because they received chemotherapy according to other protocols than the DCOG NBL2004 or the POG9640 protocols (*n* = 10), interval of more than 12 months between start of induction and ASCT (*n* = 3), less than PR at response evaluation after induction (*n* = 2), progressive disease prior to start of maintenance treatment (*n* = 3), and missing data (*n* = 1). The remaining 84 patients were included, 47 patients in the immunotherapy group and 37 in the control group.

Median follow-up of surviving patients was 7.5 years (range 4.3–18.4 years). This was 6.7 years (range 4.3–9.8 years) for the immunotherapy group and 11.6 years (range 7.5–18.4 years) for the control group. At baseline, the immunotherapy and control group were similar for clinical and pathological characteristics (Table 1). In the immunotherapy group, 39 patients (83%) completed all 6 cycles of maintenance therapy. Immunotherapy was discontinued in six patients (13%) because of progressive disease, and in two patients (4%) due to anaphylaxis. In the control group, 32 patients (86%) completed at least six cycles of isotretinoin, five patients (14%) stopped early due to progressive disease.

### 3.2. Outcome

**Figure 1:**
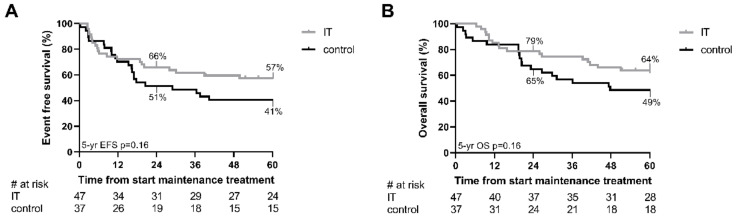
Estimated event free and overall survival for the complete cohort. (**A**,**B**) Estimated event free survival (EFS; **A**) and overall survival (OS; **B**) in months from start of the maintenance treatment, treated with immunotherapy (IT) or single-agent isotretinoin (control). Estimated survival and *p*-values are calculated by Kaplan–Meier method. Numbers at risk are given for 12 months intervals below the graphs.

At 2 years, EFS was equal to 66 ± 7% in the immunotherapy group, and to 51 ± 8% in the control group (*p* = 0.23), a 15% difference. At 5 years, EFS was equal to 57 ± 7%, and 41 ± 8% (*p* = 0.16; ∆16%) for the immunotherapy and control group, respectively (Figure 1A). At 2 years, OS was equal to 79 ± 6% and 65 ± 8% (*p* = 0.18; ∆14%), respectively. At 5 years, OS was equal to 64 ± 7% and 49 ± 8% (*p* = 0.16; ∆15%), respectively (Figure 1B).

### 3.3. Overall Survival in Patients ≥ 18 Months at Diagnosis Significantly Improved

**Figure 2:**
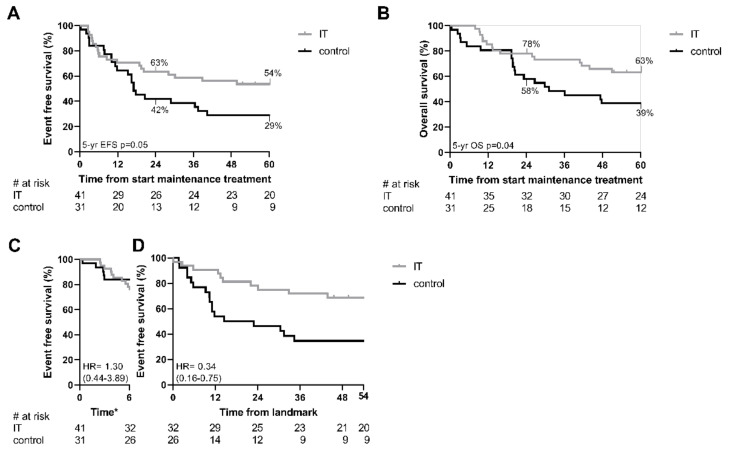
Estimated event free and overall survival of patients ≥ 18 months. (**A**,**B**) Estimated event free survival (EFS; **A**) and overall survival (OS; **B**) in months from start of the maintenance treatment for patients ≥ 18 months of age at diagnosis, treated with immunotherapy (IT) or single-agent isotretinoin (control). Numbers at risk are given for 12 month intervals below the graphs. (**C**,**D**) EFS, with a landmark at the end of treatment, 6 months after start of maintenance treatment. (**C**) shows EFS from start of maintenance treatment to landmark (6 months after start of treatment). (**D**) shows EFS from landmark until 5 years after start of maintenance treatment (54 months from landmark). Estimated survival and *p*-values are calculated by Kaplan–Meier method. Numbers at risk are given for 12 month intervals below the graphs. * Time from start maintenance therapy.

Patients older than 18 months at diagnosis are at higher risk of relapse compared to younger patients [4,5]. We performed separate analyses aimed at investigating the effect of immunotherapy in this patient group. EFS at 5 years was equal to 54 ± 8%, for the immunotherapy group and 29 ± 8% for the control group (*p* = 0.05; Figure 2A), a difference of 25%. OS at 5 years was equal to 63 ± 8% for the immunotherapy group and 39 ± 9% for the control group, an improvement of 24% (*p* = 0.04; Figure 2B). Landmark analysis was performed at the end of treatment, six months after start of maintenance treatment. Univariate Cox regression estimated on the first six months–during maintenance therapy–showed no difference in survival between the immunotherapy and control group (HR 1.30, 95% CI: 0.44–3.89; Figure 2C). From the landmark point until 5 years after start of maintenance treatment, a protective effect of the immunotherapy (HR 0.34, 95% CI 0.16–0.75; Figure 2D) was found. Addition of induction protocol to the regression model did not influence the effect of immunotherapy on survival (HR 0.36, 95% CI 0.16–0.79), and induction protocol was not associated with survival (HR 1.28, 95% CI 0.58–2.83).

## 4. Discussion

This is the first confirmation of the ANBL0032 immunotherapy protocol in a cohort outside the COG. The patients in our cohort received different induction therapy compared to the original study. The data presented here not only suggests an improved EFS and OS for high-risk neuroblastoma patients, but a long-term protective effect of immunotherapy against late events, particularly in patients ≥ 18 months of age at diagnosis.

**Figure 3:**
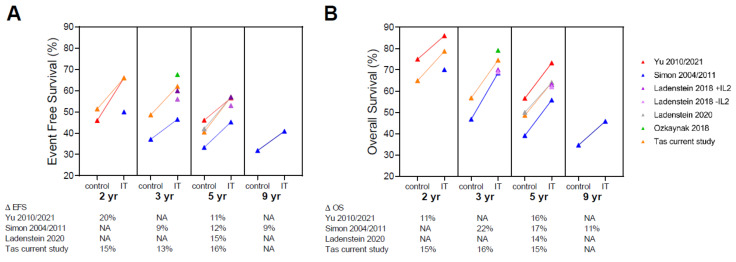
Event free and overall survival in literature. Event free survival (EFS; **A**) and overall survival (OS; **B**) of published cohorts at different times of follow-up. The X-axis shows the time of follow-up. Within each follow-up moment, the left side shows the EFS or OS of patients treated without anti-GD2 immunotherapy (control) and the right side of patients treated with immunotherapy (IT). When an article contained both patients treated with and without immunotherapy the two groups are connected by a line. Below the graph the difference (∆) in EFS or OS is given for these articles. IT: immunotherapy, yr: year, EFS: event free survival, OS: overall survival, NA: not available.

After the ANBL0032 study, immunotherapy-based maintenance treatment became standard treatment for high-risk neuroblastoma patients. Different studies have reported on the efficacy of anti-GD2 based immunotherapies (Table 2 and Figure 3). Patients in our cohort and in the COG [3,14,24], were treated according to the ANBL0032 protocol, with dinutuximab (ch14.18) [25] and alternating IL-2 and GM-CSF. In contrast, patients in the GPOH (Gesellschaft fur Padiatrische Onkologie und Hamatologie) [26,27] and SIOPEN (Société International d’Oncologie Pédiatrique European Neuroblastoma) [28,29] cohorts received dinutuximab-beta (ch14.18/CHO) [30] with/without IL-2 and no GM-CSF. Both anti-GD2 antibodies are chimeric human-mice antibodies. Dinutuximab is produced in SP2/0 cells while dinutuximab-beta is produced in CHO cells. All patients, the GPOH excepted, received concomitant isotretinoin. Despite differences in anti-GD2 antibody and concomitant drugs, the studies report an improved 5 year EFS of 11–16% and an improved OS of 14–17%. In our cohort, POG9640 or DCOG NBL2004 induction did not influence survival. In line, the pattern of improved survival for IT patients was similar for all studies, despite different induction and high-dose chemotherapy regimens (Table 2 and Figure 3).

IL-2 was given as an immunostimulant in multiple studies. Recent studies showed that IL-2 increases toxicity without improving survival and it has been deleted from all immunotherapy protocols [29,31]. The effect of the concomitant administration of isotretinoin and GM-CSF remains unclear. All studies suggest a beneficial effect on (long-term) survival. This effect seems independent of the administered anti-GD2 antibody, the induction therapy regimen, if high-dose chemotherapy is administered, and if immunostimulatory drugs are administered with the anti-GD antibody.

Interestingly, landmark analysis showed that immunotherapy had a protective effect on EFS after—but not during—maintenance therapy. This suggests that immunotherapy is more powerful in preventing late than early events. This is in line with the studies of the GPOH, who observed a more pronounced effect on long-term survival [26,27]. In contrast, the COG observed a more pronounced effect on short-term survival [3,14]. In our cohort, 68% of patients were treated with the DCOG NBL2004 protocol, which is based on the GPOH NB2004 protocol. Therefore, we wonder if the induction regimens influence this difference in prevention of late and early events. We do, however, not have the power to draw conclusions on this subject.

## 5. Conclusions

In conclusion, immunotherapy improved outcome by approximately 15% in high-risk neuroblastoma patients, with the greatest clinical benefit for patients ≥ 18 months. All studies show a sustainable effect. The effect seems most pronounced in prevention of late events, although early and some late events still occur.

## Figures and Tables

**Table 1:** Patient characteristics.

Characteristics	Control N (%)	IT N (%)	Total N (%)	Sig (*p*)
Age				0.76
<18 months	6 (16)	6 (13)	12 (14)	
≥18 months	31 (84)	41 (87)	72 (86)	
INSS stage				NA
3	3 (8)	3(6)	6(7)	
4	34 (92)	44 (94)	78 (93)	
Histology				0.43
Undifferentiated	6 (19)	11 (28)	17 (24)	
Poorly differentiated	18 (58)	24 (62)	42 (60)	
Differentiating	6 (19)	4 (10)	10 (14)	
GNB nodular	1 (3)	0 (0)	1 (1)	
Unknown	6	8	14	
MYCN				NA
Not-amplified	19 (61)	27 (61)	46 (61)	
Amplified	12 (39)	17 (39)	29 (39)	
Unknown	6	3	9	
Response prior to ASCT				0.40
CR	21 (57)	22 (47)	43 (51)	
VGPR	2 (5)	7 (15)	9 (11)	
PR	14 (38)	18 (38)	32 (38)	
Induction protocol				0.35
DCOG NBL2004	23 (62)	34 (72)	57 (68)	
POG9640	14 (38)	13 (28)	27 (32)	

IT: immunotherapy, sig: significance, NA: not applicable, GNB: ganglioneuroblastoma, ASCT: autologous stem cell transplantation, CR: complete response, (VG)PR: (very good) partial response.

**Table 2:** Literature cohorts of patients receiving immunotherapy.

Author	Yu [3,14]	Simon [26,27]	Ladenstein [28]	Ozkaynak [24]	Ladenstein [29]	Tas
Year	2010/2021	2004/2011	2020	2018	2018	Current study
Study group	COG	GPOH	SIOPEN	COG	SIOPEN	DCOG
Comparison to control group	Randomized	historical controls	historical controls	only IT	only IT	historical controls
Induction and minimal response						
Induction treatment	COG A3973	GPOH NB97	Rapid Cojec	not reported	rapid COJEC	POG9640/DCOG NBL2004
Minimal response	VGPR	NR	PR	PR	PR	PR
Immunotherapy group						
n	113	166	378	105	206/200^a^	47
HD chemotherapy	CEM	CEM/N7 courses	*BuMel*	CEM	CEM/BuMel	CEM
radiotherapy	all patients	MIBG avid masses	all patients	all patients	all patients	*all patients*
antibody	ch14.18	ch14.18/CHO	ch14.18/CHO	ch14.18	ch14.18/CHO	ch14.18
IL2	iv	no	sc/no^a^	iv	sc/no^a^	iv
GM-CSF	sc or iv	no	no	sc or iv	no	sc
RA	yes	no	yes	yes	yes	yes
Non-immunotherapy group						
n	113	69	466	0	0	37
HD chemotherapy	CEM	CEM/N7 courses	*CEM/BuMel*	NA	NA	CEM
radiotherapy	all patients	MIBG avid masses	all patients	NA	NA	*MIBG avid masses*
RA	yes	no	yes	NA	NA	yes
Reported EFS and OS						
EFS	2, 5yr	2, 3, 5, 9yr	5yr	1, 2, 3, 4, 5yr	3, 5yr	2, 5yr
OS	2, 5yr	2, 3, 5, 9yr	5yr	1, 2, 3, 4yr	3, 5yr	2, 5yr

IT: immunotherapy, COG: Children’s Oncology Group, GPOH: Gesellschaft fur Padiatrische Onkologie und Hamatologie, SIOPEN: Société International d’Oncologie Pédiatrique European Neuroblastoma, DCOG: Dutch Childhood Oncology Group, VGPR: very good partial remission, NR: no response, PR: partial remission, HD: high-dose, CEM: carboplatin/etoposide/melphalan; BuMel: busulphan/melphalan, MIBG: Iodine-123 metaiodobenzylguanidine, IL2: interleukin 2, sc: subcutaneously, iv: intravenously, GM-CSF: granulocyte macrophage colony stimulating factor, RA: retinoic acid, EFS: event free survival, yr: year, OS: overall survival. ^a^: randomization was performed with/without IL2. Discrepancies within a study between the immunotherapy and control groups are indicated by italic font.

## Data Availability

The data presented in this study are available on request from the corresponding author.
